# Supplementary material for: Identifying Molecular Modulators of the Vascular Invasion in Rectal Carcinoma: Role of ADAMTS8 and Its Co-Dependent Genes
Source: Int J Mol Sci. 2025 Jun 28;26(13):6261. doi: 10.3390/ijms26136261 (PMC12250320; doi:10.3390/ijms26136261)
Supplement: Supplementary file 1 [file ijms-26-06261-s001.zip › ijms-3661972-supplementary.pdf]

Supplementary

Table S1. Correlation of ADAMTS8 mRNA expression with other 40 pre-selected genes mRNA expression in RC dataset

Gene	Correlation Coefficient	P*	Gene	Correlation Coefficient	P*
APC	0.201	0.055	***SNAI2***	0.177	0.091
CDH1	0.073	0.487	***SNAI3***	0.139	0.187
CDKN2A	0.037	0.728	***TGFB2***	0.270	0.009
COL4A2	0.323	0.002	***TGFB3***	0.186	0.075
MDM2	0.032	0.765	***TIMP1***	-0.054	0.610
MMP10	0.223	0.033	***TWIST1***	0.093	0.379
MMP11	0.040	0.702	***VIM***	0.276	0.008
MMP13	-0.015	0.888	***ZEB1***	0.501	<0.001
MMP2	0.260	0.012	***ZEB2***	0.324	0.002
MMP3	0.246	0.018	***VCAN***	0.188	0.072
MMP7	-0.018	0.868	***ADAMTS1***	0.467	<0.001
MMP9	0.132	0.210	***ADAMTS13***	-0.113	0.285
TGFB1	0.266	0.010	***LOXL2***	0.150	0.154
TIMP2	0.296	0.004	***ECM1***	0.067	0.527
TIMP3	0.117	0.268	***MMP8***	0.151	0.150
TIMP4	-0.133	0.208	***VCAM1***	0.367	<0.001
VEGFA	-0.187	0.074	***VTN***	0.019	0.857
NOTCH1	0.113	0.283	***HIF1A***	0.199	0.058
OCLN	0.159	0.129	***VEGFB***	0.089	0.400
SNAI1	0.022	0.835	***VEGFC***	0.127	0.227

*Correlation determined using the Spearman's test

Table S2. Association between 41 pre-selected genes mRNA expression and vascular invasion in RC

Gene	Vascular invasion (N)	Mean Rank	Sum of Ranks	Z	P*
DLNA4	Negative (61)	42.33	2582.00	-2.088	0.037
	Positive (17)	29.35	499.00		
EVI2B	Negative (61)	38.62	2356.00	-0.648	0.517
	Positive (17)	42.65	725.00		
PPP1R35	Negative (61)	38.98	2378.00	-0.381	0.703
	Positive (17)	41.35	703.00		
PTGR3	Negative (61)	42.61	2599.00	-2.293	0.022
	Positive (17)	28.35	482.00		
RPL21	Negative (61)	36.41	2221.00	-2.281	0.023
	Positive (17)	50.59	860.00		
SOX4	Negative (61)	40.13	2448.00	-0.466	0.641
	Positive (17)	37.24	633.00		
ZNF3	Negative (61)	37.85	2309.00	-1.216	0.224
	Positive (17)	45.41	772.00		

*The Mann–Whitney test, $p<0.05$ was considered to indicate statistically significant

Table S3. Correlation of ADAMTS8 mRNA expression with top 7 co-depedent genes mRNA expression in RC dataset

Gene	Correlation Coefficient	P*
DLNA4	-0.091	0.429
EVI2B	0.352	0.002
PPP1R35	-0.174	0.129
PTGR3	0.219	0.054
RPL21	-0.384	0.001
SOX4	-0.288	0.011
ZNF3	-0.163	0.154

*Correlation determined using the Spearman's test

Supplementary text S1. Interactions and type of bonds of five best results for initial 23 compounds per each candidate gene.

ADAMTS8

After analyzing the structural interactions between selected compounds and ADAMTS8, it was observed that benzo[a]pyrene formed bonds with Leu332 pi-alkyl (5.16, 5.54, 5.87), pi-sigma (3.76, 3.80), His363 pi-pi t-shaped (4.82), Leu395 pi-alkyl (6.00), Phe396 pi-pi stacked (4.60, 5.30, 5.79, 5.87), pi-sigma (3.78), Lys489 pi-alkyl (5.45), Leu493 pi-alkyl (5.89), with a binding affinity of -10.2 kcal/mol. Cyanoginosin LRwith a binding affinity of -10.2 kcal/mol, exhibited interactions Glu579 H bond (3.87), Cys580 alkyl (3.77), Gly584 H bond (3.85), Leu647 alkyl (4.01), Cys666 H bond (3.40), His668 H bond (3.63), Cys679 H bonds (4.74, 4.69), Gly680 H bonds (3.28, 4.82), Ala711 H bond (4.42), Gly712 H bond (4.78), Lys760 H bond (3.60), Arg780 H bonds (2.06, 2.54, 2.81), Pro783 H bond (3.36), Asp811 H bonds (3.92, 4.69, 4.90). Doxorubicinwith a binding affinity of -9.1 kcal/mol, engaged with Met793 His182 pi-alkyl (5.51), pi-pi t-shaped (5.38), Gln183 H bond (2.80), Asp185 pi-anion (4.31), Ser186 H bond (3.50), Arg210 H bond (2.42), pi-cation (4.55, 4.64), Ala654 alkyl (3.76), Gln660 H bonds (2.38, 2.50), Cys661 H bond (3.87), alkyl (3.91), Val662 pi-alkyl (5.21, 5.62). Aflatoxin B2with a binding affinity of -8.3 kcal/mol, formed bonds with Asp185 pi-anion (3.65, 4.01), Leu205 alkyl (5.75), Arg210 H bonds (2.62, 2.65), Cys661 alkyl (4.56). Lastly, Fulvestrant participated in bonding interactions with Glu187 pi-anion (4.65), Leu205 alkyl (5.38), Pro619 alkyl (4.45), Arg622 H bonds (2.26, 3.53), Thr652 H bond (3.14), Cys661 H bond (2.84), pi-alkyl (4.13), Val662 halogen (3.93), Lys663 alkyl (3.92), with a binding affinity of -8.2 kcal/mol.

DNAL4

The interaction analysis of selected compounds with the DNAL4 revealed that benzo[a]pyrene reacted with Arg23 pi-alkyl (5.55, 5.56), Phe79 pi-pi stacked (4.30, 4.69, 4.94, 5.66, 5.89), Ile81 pi-alkyl (5.32), Tyr90 pi-pi t-shaped (4.87, 5.04),with a binding affinity of -6.9 kcal/mol. Cyanoginosin LRwith a binding affinity of -8.0 kcal/mol, reacted with Asn49 H bond (4.24), Asn50 H bonds (2.99. 3.75), Glu51 H bonds (3.53, 4.30), Ala54 alkyl (3.82), Lys55 alkyl (4.26), Lys58 alkyl (3.80, 3.83), Trp69 H bond (4.59), His70 H bond (2.00), Val71 H bonds (4.51, 4.56), alkyl (4.30), Ile73 H bonds (4.06, 4.86), Glu75 H bond (2.82), Gly76 H bond (4.64), Gly78 H bond (3.21). Doxorubicin reacted with Glu51 H bond (3.27), Ala54 pi-alkyl (5.10, 5.21), alkyl (3.72), Lys55 alkyl (5.22), Val72 pi-alkyl (4.74, 5.29, 5.41), Glu75 H bond (2.41), Gly76 H bond (2.72), with a binding affinity of -6.7 kcal/mol. Fulvestrant reacted with Glu51 halogen (3.13), Ala54 alkyl (3.80), halogen (3.13, 3.75), Lys55 alkyl (4.17), Lys58 alkyl (4.79), His70 H bond (2.00), pi-sulfur

(4.63), Val72 alkyl (4.46, 4.58, 5.27, 5.80), Ile73 alkyl (4.89), Phe77 pi-alkyl (5.61), with a binding affinity of -6.5 kcal/mol. Progesteronewith a binding affinity of -6.9 kcal/mol, reacted with His24 H bond (2.78), pi-alkyl (5.62), Phe79 pi-alkyl (4.69, 4.85, 5.39), Tyr90 pi-alkyl (5.60, 5.85).

EVI2B

After conducting an analysis of the structural interactions between selected compounds and the EVI2B, it was observed that benzo[a]pyrene reacted with Ile222 pi-pi alkyl (4.98, 5.25, 5.78), Trp225 pi-pi stacked (3.62, 3.79, 3.88, 3.91, 3.96, 4.18, 4.55, 4.57, 5.10) Lys226 pi-alkyl (5.08),with a binding affinity of -7.8 kcal/mol. Cyanoginosin LR reacted with Leu8 pi-alkyl (5.95), Phe11 pi-alkyl (4.81, 4.93, 5.17), pi-pi t-shaped (4.89), Leu15 alkyl (4.78, 5.52), Phe19 pi-alkyl (5.75), Ala205 H bond (3.33), Ile206 H bond (4.64), alkyl (4.48), Gly209 H bond (4.86), Val210 H bonds (4.40, 4.42), Thr213 H bonds (3.46, 3.65), Leu216 alkyl (4.82), Val217 alkyl (4.63), Ile220 pi-sigma (3.56),with a binding affinity of -8.3 kcal/mol. Doxorubicinwith a binding affinity of -6.1 kcal/mol, reacted with Lys226 pi-alkyl (5.15), Arg229 H bond (2.13, 3.22), Val232 pi-alkyl (3.90, 5.26), Asn234 H bond (3.16), Asp235 H bond (1.94), Trp238 pi-alkyl (4.50). Estradiolwith a binding affinity of -6.6 kcal/mol, reacted with Ile222 pi-alkyl (5.07), alkyl (5.16), Trp225 pi-pi stacked (3.94, 3.96), pi-alkyl (3.98, 4.40, 4.43, 4.51, 5.81), Lys226 alkyl (4.27, 5.20, 5.51). Progesteronewith a binding affinity of -6.9 kcal/mol, reacted with Trp225 pi-alkyl (3.92, 4.74, 4.81), pi-sigma (3.52, 3.56), Leu228 alkyl (4.47, 5.77), Arg229 H bond (5.98).

PPP1R35

Upon analyzing the structural interactions of selected compounds with PPP1R35, it was shown that aflatoxin B2 reacted with Phe215 pi-pi stacked (5.17, 5.56), pi-alkyl (4.23), Leu231 alkyl (4.26), Arg234 H bond (3.57), pi-alkyl (3.94, 4.48), alkyl (4.82), Pro235 H bond (3.46), with a binding affinity of -6.4 kcal/mol. Benzo[a]pyrene reacted with Phe215 pi-pi stacked (4.13, 4.16, 5.07, 5.42, 5.61), Leu231 pi-alkyl (5.18), Arg234 pi-alkyl (4.37, 5.05, 5.23), with a binding affinity of -7.3 kcal/mol. Cyanoginosin LR reacted with Ile207 alkyl (3.71), Tyr216 pi-alkyl (5.23), Glu217 H bond (3.24), Arg236 H bonds (2.69, 2.83), Leu243 alkyl (4.13, 4.94), pi-sigma (3.54), Met244 H bond (2.47), with a binding affinity of -9.2 kcal/mol. Doxorubicin reacted with Thr213 H bond (3.52), Phe215 H bond (3.46), pi-pi t-shaped (5.11, 5.15), Leu231 pi-sigma (3.90), Leu233 H bond (3.38), Pro235 H bond (2.50),with a binding affinity of -7.0 kcal/mol. Estradiolwith a binding affinity of -6.4 kcal/mol, reacted with Leu223 H bond (2.10), Leu226 alkyl (4.50), Pro228 alkyl (5.04), Leu229 alkyl (4.48, 5.07), pi-sigma (3.65).

PTGR3

The interaction analysis of selected compounds with PTGR3 revealed that aflatoxin B2 reacted with Ala67 pi-alkyl (4.63), pi-sigma (3.99), Ala171 pi-alkyl (5.16), alkyl (4.35), Thr176 pi-sigma (3.68), Ser238 H bond (3.55), Ile263 pi-alkyl (4.64), alkyl (5.21), Met347 alkyl (5.00),with a binding affinity of -9.0 kcal/mol. Benzo[a]pyrenewith a binding affinity of -9.9 kcal/mol, reacted with Ala67 pi-alkyl (5.35), Tyr77 pi-pi t-shaped (5.69), Phe89 pi-pi t-shaped (4.76), Val146 pi-alkyl (5.28, 5.39), Thr150 H bond (3.46), Leu296 pi-alkyl (5.48). Cyanoginosin LRwith a binding affinity of -9.8 kcal/mol reacted with Arg76 H bond (3.36), Tyr77 pi-alkyl (4.50), Phe89 pi-pi stacked (3.84), Val146 pi-alkyl (5.15), Val259 alkyl (5.23), Thr270 H bond (3.32), Gly293 H bond (3.37), Phe295 pi-alkyl (4.81, 5.23, 5.31), Leu296 alkyl (4.91). Doxorubicin reacted with Tyr152 H bond (3.73), Lys156 pi-sigma (3.93), Leu161 H bond (2.82), Lys186 alkyl (3.96), Gln267 H bond (3.47), Leu272 H bonds (2.23, 2.55), His309 H bonds (3.28, 3.58), pi-alkyl (5.02),with a binding affinity of -9.0 kcal/mol. Fulvestrantwith a binding affinity of -9.0 kcal/mol reacted with Tyr77 pi-sulfur (4.27), Phe89 pi-pi t-shaped (4.72), Met115 pi-alkyl (5.49), Val146 pi-alkyl (4.83), alkyl (5.00, 5.27, 5.46), Val259 halogen (3.17, 3.49), Ile260 halogen (3.16), Gly293 H bond (3.61), Phe294 H bond (2.88), halogen (3.00, 3.05, 3.55, 3.60), Phe295 pi-alkyl (5.38), Leu296 pi-alkyl (5.48), alkyl (5.11).

RPL21

Analysis of structural interactions between selected compounds and RPL21 showed that benzo[a]pyrene with a binding affinity of -6.8 kcal/mol, reacted with Trp125 pi-pi stacked (3.62, 3.71, 3.92, 4.09, 4.18, 4.22, 4.58, 5.55), Leu128 pi-alkyl (5.50), Lys129 pi-alkyl (4.82). Cyanoginosin LRwith a binding affinity of -7.4 kcal/mol, reacted with Asn3 H bond (4.46), Arg9 H bonds (2.09, 2.14, 2.19, 2.49, 4.51), Gly10 H bond (3.38), Tyr13 pi-alkyl (4.92), Met14 pi-alkyl (5.07), Lys55 H bonds (2.24, 2.44, 4.80), Gln79 H bonds (2.94, 4.47). Doxorubicinwith a binding affinity of -6.5 kcal/mol, reacted Lys7 H bond (2.46), Met52 alkyl (4.88), Asn90 H bonds (2.30, 2.44, 3.05), Ile89 pi-alkyl (5.47, 5.77). Estradiol reacted with Ile39 H bond (3.03), pi-alkyl (5.20), His98 pi-alkyl (5.67), Arg102 pi-alkyl (102), Asp103 pi-anion (3.81), Leu106 pi-alkyl (5.83), alkyl (4.54, 5.62),with a binding affinity of -6.8 kcal/mol. Progesteronewith a binding affinity of -6.6 kcal/mol, reacted with Arg17 H bonds (2.21, 2.31), Arg32 alkyl (5.61), Met45 alkyl (5.43), Lys97 alkyl (4.05, 4.41, 5.38).

SOX4

Upon investigating the structural interactions between selected compounds and SOX4, it was observed that aflatoxin B2with a binding affinity of -6.6 kcal/mol, reacted with Asp47 H bond (3.35), Pro49 alkyl (5.42), Trp51 H bond (2.68), Arg114 H bond (2.37), Ser466 H bond (2.89). Benzo[a]pyrene reacted with Leu100 pi-alkyl (5.62), Phe472 pi-pi stacked (3.70, 4.01, 4.42, 5.30),with a binding affinity of -7.8 kcal/mol.

Cyanoginosin LRwith a binding affinity of -8.0 kcal/mol, reacted with Pro107 H bond (4.56), Arg110 H bonds (2.52, 3.83), Arg114 alkyl (3.72, 4.37), Trp462 pi-alkyl (5.38), Leu463 alkyl (5.07), Asn469 H bond (3.12), Leu470 alkyl (4.62), Phe472 pi-pi stacked (4.25). Doxorubicinwith a binding affinity of -7.2 kcal/mol, reacted with Phe66 pi-alkyl (5.77), Met67 alkyl (3.80), Leu115 alkyl (4.58), Ser465 H bond (2.56). Progesteronewith a binding affinity of -6.9 kcal/mol, reacted with Arg114 alkyl (5.58), Lys118 H bond (2.82), alkyl (4.93), Trp462 pi-alkyl (3.88, 4.24, 4.57, 4.83, 5.40, 5.88).

ZNF3

A detailed analysis of the structural interactions between selected compounds and ZNF3 revealed that benzo[a]pyrene reacted with Arg62 pi-alkyl (5.08), Trp65 pi-pi stacked (3.71, 3.80, 3.81, 3.87, 4.07, 4.18, 4.63, 4.86, 5.14), Arg77 pi-alkyl (5.19),with a binding affinity of -9.1 kcal/mol. Cyanoginosin LRwith a binding affinity of -10.1 kcal/mol, reacted with Val58 H bond (4.59, 4.60), Tyr59 pi-alkyl (4.90, 5.20), Phe60 H bond (3.30), Trp65 H bond (3.02), Tyr76 H bond (2.13), Arg77 H bond (2.23), Phe116 pi-alkyl (5.56), Ile120 pi-alkyl (5.31), alkyl (4.78, 4.83), pi-sigma (3.51), Gly123 H bond (3.39), Leu124 pi-alkyl (5.04), alkyl (4.66, 4.68, 5.23), Lys127 alkyl (4.71). Doxorubicin reacted with Phe60 H bonds (2.75, 2.77), Tyr76 H bond (1.94), Leu81 pi-alkyl (5.43), alkyl (4.76), Phe116 pi-pi t-shaped (5.28), Ile120 pi-alkyl (5.14), alkyl (3.71), Gln122 H bond (3.00),with a binding affinity of -8.1 kcal/mol. Estradiol reacted with Val58 alkyl (4.80, 5.80), Phe60 pi-alkyl (5.90), Gln72 H bond (2.07), Leu75 pi-alkyl (5.02), amide-pi stacked (4.55), Tyr76 pi-alkyl (5.46), Val79 pi-alkyl (5.41), alkyl (3,80, 4.80, 5.02), Ser185 H bond (3.58),with a binding affinity of -8.2 kcal/mol. Fulvestrant reacted with Phe60 H bond (2.19), Trp65 pi-alkyl (4.20, 4.64, 5.63), Tyr76 pi-alkyl (5.13, 5.32), Arg77 alkyl (4.97), halogen (3.81), pi-cation (4.14), Met80 alkyl (4.42, 5.71), halogen (3.33, 3.33, 3.53), Leu81 alkyl (4.37), Gly123 H bond (2.67),with a binding affinity of -7.9 kcal/mol.

Supplementary text S2. Interactions and type of bonds of 10 best result for similar 9661 compounds per each candidate gene.

ADAMTS8

After analyzing the structural interactions between selected compounds and ADAMTS8, it was observed that dibenzo(a,e)pyrene (9126) formed bonds with Leu332 pi-sigma (3.51, 3.52, 3.59), pi-alkyl (5.23, 5.71, 5.77), Glu353 pi-anion (4.98), His363 pi-pi t-shaped (4.80), Leu395 pi-alkyl (5.41), Phe396 (4.70, 5.28), Leu493 pi-alkyl (5.89),with a binding affinity of -11.2 kcal/mol. 148413with a binding affinity of -11.3 kcal/mol, exhibited interactions with Leu332 pi-sigma (3.43, 3.80, 3.98), pi-alkyl (5.19, 5.42, 5.75), Glu353 pi-anion (4.63), His363 pi-pi t-shaped (4.93), Leu395 pi-alkyl (4.66, 5.81, 5.84), Phe396 H bond (2.95), pi-

alkyl (4.11), pi-pi stacked (5.07, 5.22), Lys489 pi-alkyl (5.43). 169380 engaged with Leu332 pi-sigma (3.45, 3.95), pi-alkyl (4.89, 4.90, 5.59, 5.69), Glu353 pi-anion (4.82), His363 pi-pi t-shaped (4.77), Leu395 pi-alkyl (5.46, 5.60), Phe396 pi-pi stacked (4.35, 5.29, 5.45, 5.73), Leu493 pi-alkyl (5.98),with a binding affinity of -11.5 kcal/mol. 626153 with a binding affinity of -11.2 kcal/mol, formed bonds with Leu332 pi-sigma (3.49, 3.95), pi-alkyl (5.22, 5.62, 5.66), H Bond (3.22) Gly333 H bond (3.30), Glu353 pi-anion (4.87), Thr360 H bond (3.34), halogen bond (3.87), His363 pi-alkyl (4.77), Glu364 halogen bond (3.65, 3.88), Phe396 pi-pi stacked (4.39, 5.35, 5.46, 5.76), Leu493 pi-alkyl (5.94). 12588587 participated in bonding interactions with Asp330 halogen bond (3.15, 3.38), Leu332 pi-sigma (3.63, 3.87), pi-alkyl (5.52, 5.56), Gln356 halogen bond (3.34), His363 pi-pi t-shaped (4.77), Glu364 halogen bond (3.55), Pro394 halogen bond (3.42, 3.76), Leu395 pi-alkyl (5.82), Phe396 pi-pi stacked (5.67), Asn490 halogen bond (3.06),with a binding affinity of -12.3 kcal/mol. 14274984 reacted with Leu332 pi-sigma (3.42, 3.95), pi-alkyl (4.87, 4.89, 5.57, 5.67), Glu353 pi-anion (4.92), His363 pi-pi t-shaped (4.76), Leu395 pi-alkyl (5.63), Phe396 H bond (2.99), pi-pi stacked (4.35, 5.31, 5.44, 5.74), Lys489 alkyl bond (3.90), Leu493 pi-alkyl (5.99),with a binding affinity of -11.6 kcal/mol. 15001195with a binding affinity of -11.3 kcal/mol, reacted with Val249 pi-sigma (3.92), pi-alkyl (4.64, 5.63, 5.86), Arg252 pi-alkyl (5.23, 5.47, 5.63), Ile253 pi-alkyl (4.46, 4.80, 5.76), Leu355 pi-alkyl (5.30), His359 pi-pi t-shaped (5.18, 5.34), pi-cation (4.20, 4.76), Leu399 pi-alkyl (5.35, 5.54, 5.79), Pro404 pi-alkyl (4.88), Pro440 pi-alkyl (5.13), Asp497 pi-anion (3.69, 3.99, 4.31). 101392782 reacted with Leu332 pi-sigma (3.40), pi-alkyl (4.72, 5.25, 5.68), Phe396 pi-alkyl (5.31), Lys489 pi-alkyl (4.89, 5.67), Leu493 (5.49), with a binding affinity of -11.4 kcal/mol. 129716757with a binding affinity of -11.6 kcal/mol, reacted with Leu332 pi-sigma (3.59, 3.62, 3.76), pi-alkyl (5.19, 5.90), His363 H bond (2.94), pi-pi t-shaped (4.89), His373 H bond (2.97), Phe396 pi-pi stacked (4.71, 5.23), Leu493 pi-alkyl (5.86). Lastly, 129805998 participated in bonding interactions with Asp330 halogen bond (3.31), Leu332 pi-sigma (3.61, 3.85), pi-alkyl (5.46, 5.59), Gln356 halogen bond (3.23), His363 pi-pi t-shaped (5.08), Glu364 halogen bond (3.31, 3.55), Pro394 halogen bond (3.53, 3.66), Leu395 pi-alkyl (5.90), Phe396 pi-pi stacked (5.49), Asn490 halogen bond (2.95),with a binding affinity of -11.3 kcal/mol.

DNAL4

The interaction analysis of selected compounds with *DNAL4* revealed that dibenzo(a,e)pyrene (9126)with a binding affinity of -7.7 kcal/mol, reacted with Arg23 pi-alkyl (5.06), Phe79 pi-pi stacked (4.42, 4.76, 4.84), Ile81 pi-alkyl (5.57), Tyr90 pi-pi t-shaped (5.11). 12588587with a binding affinity of -7.9 kcal/mol, reacted with Arg23 H bonds (2.59, 2.99, 3.03), halogen bond (3.90), pi-alkyl (5.50), Phe79 pi-pi stacked (4.93, 5.71, 5.81), halogen bonds (2.81, 2.98), Glu80 H bond (3.45), Ile81 H bonds (2.29, 2.86), halogen bonds (2.80. 3.42), His83 H bonds (2.93, 3.59), Leu88 pi-alkyl (5.69), Tyr90 pi-pi t-shaped (4.93). 14274984 reacted with Arg23 pi-alkyl (5.24, 5.45), Phe79 pi-alkyl (4.58), pi-pi stacked (4.35, 4.69, 5.28,

5.91), Tyr90 pi-pi t-shaped (4.71, 5.13, 5.32). 15001195 reacted with Arg23 pi-alkyl (5.12, 5.52), Phe79 pi-pi stacked (4.40, 5.04, 5.12), Ile81 pi-alkyl (5.2), Leu88 pi-alkyl (5.59, 5.89), Tyr90 pi-pi t-shaped (4.87, 5.01, 5.30),with a binding affinity of -7.6 kcal/mol. 56664790 reacted with Ala54 pi-alkyl (4.61), His70 H bonds (1.87, 2.53), Val72 pi-alkyl (5.31, 5.45, 5.80), Ile73 H bond (2.44), Trp102 pi-pi t-shaped (5.27), with a binding affinity of -7.7 kcal/mol. 101023804with a binding affinity of -7.8 kcal/mol, reacted with Lys55 alkyl (4.40), Lys58 alkyl (5.80), His70 H bond (2.73), Val72 pi-alkyl (5.26, 5.52), alkyl (5.45), Gly78 H bond (3.85), Trp102 pi-pi t-shaped (5.16, 5.30). 101392782with a binding affinity of -7.9 kcal/mol, reacted with Arg23 pi-alkyl (5.45), alkyl (5.79), Phe79 pi-alkyl (4.55), pi-pi stacked (4.54, 4.68, 5.59), Ile81 pi-alkyl (5.19), Tyr90 pi-alkyl (5.37), pi-pi t-shaped (4.89). 102060712 participated in bonding interactions with Asn50 H bond (2.41), Glu51 H bond (3.78), pi-anion (3.47), Lys55 pi-alkyl (4.84), His70 H bond (2.68), Val71 H bond (2.21), Val72 pi-alkyl (5.27, 5.70), alkyl (5.33), Ile73 H bond (2.32), Trp102 pi-pi t-shaped (5.15, 5.33),with a binding affinity of -7.9 kcal/mol. 129628257with a binding affinity of -7.6 kcal/mol, reacted with Arg15 alkyl (4.94), Leu16 pi-alkyl (5.83), alkyl (5.73), Phe19 pi alkyl (4.70), pi-pi stacked (3.71, 3.82, 4.14). 129716757 reacted with Arg23 pi-alkyl (5.35, 5.49), His24 H bond (2.70), Phe79 pi-pi stacked (4.31, 4.92, 5.08, 5.91, 5.96), Ile81 pi-alkyl (5.71), His83 H bond (2.67), Tyr90 pi-pi t-shaped (4.79, 5.04, 5.39),with a binding affinity of -8.2 kcal/mol.

EVI2B

After conducting an analysis of the structural interactions between selected compounds and *EVI2B*, it was observed that dibenzo(a,e)pyrene (9126) reacted with Ile222 pi-alkyl (5.10, 5.54), Trp225 pi-pi stacked (3.57, 3.67, 3.92, 4.04, 4.07, 4.29, 4.31, 4.39, 4.87, 5.30) Lys226 pi-alkyl (5.21),with a binding affinity of -8.1 kcal/mol. 159823with a binding affinity of -8.2 kcal/mol, reacted with Ile222 pi-alkyl (4.99, 5.31, 5.88), Trp225 pi-pi stacked (3.62, 3.74, 3.91, 3.94, 4.03, 4.28, 4.45, 5.24), Lys226 alkyl (4.42), Arg229 H bond (2.58). 160349with a binding affinity of -8.3 kcal/mol, reacted with Ile222 pi-alkyl (5.07, 5.35, 5.83), Trp225 pi-pi stacked (3.65, 3.81, 3.84, 3.90, 3.94, 4.07, 4.31, 4.64, 4.70, 4.96, 5.41) Lys226 pi-alkyl (4.95). 187315with a binding affinity of -8.3 kcal/mol, reacted with Ile222 pi-alkyl (5.00, 5.24, 5.8), Trp225 pi-pi stacked (3.63, 3.81, 3.88, 3.91, 3.97, 4.17, 4.56, 4.64, 5.10), Lys226 pi-alkyl (5.07), Arg229 H bond (2.80). 14274984 reacted with Ile222 pi-alkyl (4.90, 5.29, 5.66), Trp225 pi-pi stacked (3.62, 3.70, 3.93, 3.95, 4.01, 4.06, 4.32, 4.41, 4.42, 4.88, 5.26), pi-alkyl (5.22), Lys226 pi-alkyl (5.35),with a binding affinity of -8.1 kcal/mol. 15001195with a binding affinity of -8.2 kcal/mol, reacted with Ile222 pi-alkyl (5.05, 5.37, 5.56), Trp225 pi-pi stacked (3.63, 3.78, 3.88, 3.91, 4.03, 4.18, 4.24, 4.47, 5.19, 5.36), Lys226 pi-alkyl (4.73). 23617881with a binding affinity of -8.1 kcal/mol, reacted with Ile7 pi-alkyl (4.81, 4.95), Leu10 pi-alkyl (5.71), Phe11 pi-pi t-shaped (4.92, 5.14), Leu216 pi-alkyl (5.40, 5.66), Ile219 alkyl (4.98), Ile220 pi-alkyl (4.15, 5.35, 5.53), alkyl (4.69), Val223 pi-alkyl (4.90), alkyl (3.76), Leu224 pi-alkyl (5.19). 129628257

reacted with Ile221 alkyl (5.81), Trp225 pi-pi stacked (3.78, 3.79, 3.80, 4.09, 4.38, 4.96) pi-alkyl (4.87, 5.37), Arg229 pi-alkyl (5.27, 5.47),with a binding affinity of -8.4 kcal/mol. 129716757with a binding affinity of -8.7 kcal/mol, reacted with Ile222 pi-alkyl (5.10, 5.64), Trp225 pi-pi stacked (3.66, 3.76, 3.90, 3.94, 3.97, 4.30, 4.44, 4.54, 5.17), Lys226 H bonds (2.34, 2.63), pi-alkyl (5.10), Arg229 H bonds (2.78, 2.99). Lastly, 129762518 participated in bonding interactions with Ile221 pi-alkyl (5.02), Leu224 pi-alkyl (5.74), Trp225 H bond (2.73), pi-pi stacked (3.90, 4.03, 4.06, 4.19, 5.00, 5.02, 5.67), Leu228 pi-alkyl (5.45), Arg229 H bonds (1.97, 2.57), pi-alkyl (5.95)with a binding affinity of -8.3 kcal/mol.

PPP1R35

Upon analyzing the structural interactions of selected compounds with *PPP1R35*, it was shown that dibenzo(a,e)pyrene (9126) reacted with Phe215 pi-pi stacked (3.95, 4.80. 5.00, 5.18, 5.66), Leu231 pi-alkyl (5.24), Gln232 pi-sigma (3.98), Arg234 pi-alkyl (4.18, 4.23, 4.45, 5.53, 5.80), with a binding affinity of -8.3 kcal/mol. 153936 reacted with Ile207 H bond (2.21), Leu208 pi-alkyl (5.49), Arg234 pi-alkyl (4.05, 4.74, 4.76), Leu243 pi-alkyl (5.66), with a binding affinity of -8.0 kcal/mol. 12588587 reacted with Phe215 pi-pi stacked (5.25, 5.79, 5.87), Arg230 halogen (3.93), Gln232 H bonds (2.86, 3.02), halogen (3.01, 3.10) Arg234 pi-alkyl (3.77, 4.44, 4.66, 5.56), halogen (3.44), Arg236 pi-alkyl (5.25), Pro237 H bond (3.26, 3.98), Asp240 halogen (3.33), with a binding affinity of -7.9 kcal/mol. 14274984with a binding affinity of -8.1 kcal/mol reacted with Phe215 pi-pi stacked (4.02, 4.31, 5.00, 5.07, 5.77), Leu231 pi-alkyl (5.47), alkyl (3.52), Arg234 pi-alkyl (4.16, 4.50, 5.13, 5.33, 5.60). 23617881 reacted with Leu223 pi-alkyl (5.89), Leu226 pi-alkyl (5.02), Pro228 pi-alkyl (4.53, 5.15), Leu229 pi-alkyl (5.21, 5.62, 5.74), alkyl (5.23), pi-sigma (3.58, 3.88, 3.94), Leu231 pi-alkyl (5.86), alkyl (4.36), with a binding affinity of -8.4 kcal/mol. 23621448with a binding affinity of -9.0 kcal/mol, reacted with Phe215 pi-pi stacked (3.99, 4.44, 5.05, 5.36), pi-alkyl (4.82), Leu231 pi-alkyl (5.23), alkyl (3.87), Gln232 pi-sigma (3.81), Arg234 pi-alkyl (3.95, 4.03, 5.12, 5.16, 5.77). 71452697 reacted with Thr213 H bonds (2.04, 2.87, 3.76, 3.88), Phe215 pi-pi t-shaped (5.31), Leu231 pi-alkyl (4.54), Leu233 H bond (3.49), Arg234 H bonds (2.70, 2.93), Arg236 H bonds (2.37, 3.19), Leu243 alkyl (5.23), halogen (3.98), Met244 H bond (2.76), with a binding affinity of -8.1 kcal/mol. 102224786with a binding affinity of -8.0 kcal/mol, reacted with Thr213 H bond (3.74), Leu214 H bond (3.55), Phe215 H bond (2.68), pi-pi t-shaped (5.23, 5.51), Leu231 pi-alkyl (4.47), Leu233 H bond (3.39), Arg234 H bond (2.92), alkyl (3.99, 5.87), Asp240 H bonds (2.25, 2.52, 3.07), Leu243 alkyl (5.06). 129628257 reacted with Phe215 pi-alkyl (5.0), Leu231 pi-alkyl (5.09, 5.10), Arg234 alkyl (4.59, 5.56), with a binding affinity of -8.1 kcal/mol. Lastly, 129853608 participated in bonding interactions with Phe215 pi-alkyl (4.93), pi-pi stacked (4.03, 4.78, 5.49), Leu231 pi-alkyl (5.05, 5.15), Arg234 pi-alkyl (3.98), with a binding affinity of -8.1 kcal/mol.

PTGR3

The interaction analysis of selected compounds with *PTGR3* revealed that dibenzo(a,e)pyrene (9126)with a binding affinity of -10.9 kcal/mol, reacted with Ala67 pi-pi alkyl (4.10, 4.37, 4.42, 4.48), Ala171 pi-pi alkyl (4.38, 5.11), Thr176 pi-sigma (3.76), Ile263 pi-alkyl (4.68), pi-sigma (3.83), Met347 pi-alkyl (5.15, 5.17), pi-sigma (3.43), Lys355 pi-alkyl (5.15), Ile356 pi-alkyl (5.81). 42890with a binding affinity of -11.5 kcal/mol, reacted with Ala67 pi-alkyl (4.14, 4.21), Ala171 pi-alkyl (5.02), Val259 alkyl (5.25), Ile260 H bond (3.22), Gly261 H bonds (3.54, 3.69), Phe262 pi-alkyl (4.99), Ile263 pi-alkyl (4.66). 186437with a binding affinity of -11.0 kcal/mol, reacted with Ala67 pi-alkyl (4.07, 4.58, 4.61, 4.80), Thr150 H bond (2.28), Ala171 pi-alkyl (4.69), Gly174 H bonds (3.90), Gly175 H bond (3.02), Thr176 pi-sigma (3.57), Ile260 pi-alkyl (5.83), Ile263 pi-alkyl (4.88, 5.07), Met347 pi-alkyl (5.21, 5.76), Asn352 H bonds (2.37). 10099105 reacted with Ala67 pi-alkyl (4.01, 4.18, 5.20), Ala171 pi-alkyl (5.09), Val259 alkyl (5.18), Ile260 H bonds (2.97, 3.44), Gly261 H bond (3.39), Phe262 pi-alkyl (4.80), Ile263 pi-alkyl (4.74), Tyr266 H bond (2.40), Leu296 H bond (2.89), with a binding affinity of -11.6 kcal/mol. 12588587 reacted with Asn66 halogen (3.07, 3.41), Ala67 pi-alkyl (3.69, 3.98, 4.79, 5.02), Ala171 pi-alkyl (4.69), Gly174 H bond (3.63), halogen (3.95), Gly175 H bonds (2.19, 3.01), Ser238 H bond (3.78), halogen (3.07, 3.28), Ile260 halogen (2.83), Gly261 H bond (3.33, 3.42), Ile263 pi-alkyl (5.27, 5.52, 5.92), Met347 pi-alkyl (4.98, 5.54), Asn352 H bonds (2.18, 2.48), halogen (3.26), with a binding affinity of -11.8 kcal/mol. 13553135 reacted with Tyr152 pi-pi t-shaped (4.85), Leu155 pi-alkyl (5.83), Lys156 pi-pi alkyl (5.09, 5.43), Leu161 pi-alkyl (4.94), Leu183 pi-alkyl (4.53, 5.29, 5.44), Lys186 pi-pi alkyl (4.20, 5.10, 5.77), alkyl (4.27), Ala187 pi-pi alkyl (4.07, 4.55, 4.74), Pro269 pi-alkyl (5.25), His309 pi-pi stacked (5.11), Met313 pi-sulfur (4.83), with a binding affinity of -11.2 kcal/mol. 23617881 reacted with Ala67 pi-alkyl (4.12, 4.17, 4.22, 5.25), pi-sigma (3.52), Ala171 pi-alkyl (4.39, 4.57) Ile260 alkyl (5.84), Ile263 pi-alkyl (4.93, 5.82), pi-sigma (3.89), Met347 pi-alkyl (4.95), pi-sigma (3.50), pi-sulfur (4.49), Lys355 pi-alkyl (5.28), with a binding affinity of -11.1 kcal/mol. 129628257with a binding affinity of -11.3 kcal/mol, reacted with Tyr152 pi-alkyl (4.91), Leu155 alkyl (5.47), Lys156 pi-alkyl (3.91), pi-sigma (3.65, 3.98), Leu161 pi-alkyl (5.32), alkyl (5.96), Leu183 alkyl (4.42, 5.40), Lys186 alkyl (5.74), His309 pi-alkyl (5.64). 129716757with a binding affinity of -11.4 kcal/mol, reacted with Ala67 pi-alkyl (4.31, 4.48, 4.91), Ala171 pi-alkyl (4.45), Gly175 H bond (2.21), Thr176 pi-sigma (3.72, 3.76), Ile260 pi-alkyl (5.90, 5.93), Ile263 pi-alkyl (4.56), Asn352 H bond (2.40). Lastly, 129805998with a binding affinity of -11.0 kcal/mol, participated in bonding interactions with Asn66 halogen (3.11, 3.44), Ala67 H bonds (2.45, 2.59), pi-alkyl (4.62), Ser68 H bond (3.05), Phe89 pi-pi t-shaped (5.55), Val146 pi-alkyl (4.76, 5.31, 5.96), Ser238 H bonds (3.80), Leu296 H bonds (2.38, 2.82), pi-alkyl (5.22), halogen (3.76), Asn297 H bonds (2.48, 3.02), halogen (3.05, 3.36).

RPL21

Analysis of structural interactions between selected compounds and *RPL21* showed that dibenzo(a,e)pyrene (9126) reacted with Lys35 (4.63), Asp38 pi-anion (3.33, 3.66, 3.77, 4.30), Ile39 H bond (3.04), pi-alkyl (5.19), Arg102 pi-alkyl (4.80), Asp103 pi-anion (3.73), Leu106 pi-alkyl (5.31, 5.77), pi-sigma (3.56), with a binding affinity of -8.2 kcal/mol. 12588587 reacted with Tyr30 halogen (3.71), Arg32 halogen (3.61, 3.88), Ile33 H bond (3.84), Tyr34 H bond (2.61), halogen (3.11), Thr68 halogen (3.82), Gln69 halogen (2.99, 3.24), His70 H bonds (3.11, 3.44), halogen (3.24, 3.43), Ala71 halogen (3.27), Ile93 pi-alkyl (4.90. 5.38, 5.57), with a binding affinity of -8.3 kcal/mol. 14274984with a binding affinity of -7.6 kcal/mol, reacted with Trp125 pi-pi stacked (3.68, 3.87, 3.89, 3.93, 4.03, 4.39, 4.75, 4.80, 5.23, 5.41), Leu128 pi-pi alkyl (5.43, 5.85), Lys129 alkyl (3.90), pi-alkyl (5.13). 23617881with a binding affinity of -8.2 kcal/mol, reacted with Lys35 alkyl (4.17), Asp38 pi-anion (3.55, 3.71, 3.77, 4.72), Ile39 H bond (3.16), pi-alkyl (5.27), His98 pi-pi stacked (4.15), Arg102 pi-alkyl (4.77), Asp103 pi-anion (3.59), Leu106 pi-alkyl (5.30, 5.99), pi-sigma (3.68). 23621448 reacted with Lys116 pi-alkyl (5.04), Trp125 pi-pi stacked (3.71, 3.83, 3.86, 4.11, 4.28, 4.36, 5.23, 5.44, 5.75) pi-alkyl (4.19), Leu128 pi-alkyl (5.61, 5.92), Lys129 pi-alkyl (4.83), with a binding affinity of -8.0 kcal/mol. 70695534 reacted with Ile33 pi-alkyl (5.58), Val67 pi-alkyl (5.40), Gln69 H bond (2.43), Arg92 H bond (2.43), Ile93 pi-alkyl (5.10, 5.47, 5.88), Glu94 H bond (2.10), with a binding affinity of -7.9 kcal/mol. 90678391with a binding affinity of -7.8 kcal/mol, reacted with Arg17 H bonds (2.39, 2.40), Arg32 alkyl (5.44), Lys43 alkyl (3.73, 3.77), Met45 alkyl (4.13, 5.65), Lys97 alkyl (3.89, 4.03, 4.49, 4.95). 101023800 reacted with Pro26 pi-alkyl (4.22, 4.32), Ala28 pi-alkyl (5.60, 5.61), Thr29 pi-sigma (3.96), Arg32 H bond (2.00), Lys43 alkyl (3.98), Lys97 alkyl (4.17), with a binding affinity of -7.7 kcal/mol. 101346118with a binding affinity of -7.9 kcal/mol, reacted with Arg17 H bonds (1.88, 2.49), Arg32 alkyl (5.66), Lys43 H bond (2.93), Met45 alkyl (5.82), Lys97 alkyl (3.89, 4.32, 5.29). Lastly, 129628257 participated in bonding interactions with Lys116 alkyl (4.62, 5.34), Ala119 alkyl (5.05), Lys120 alkyl (5.48), Trp125 (3.76, 3.83, 3.93, 4.44, 4.64, 5.07) Leu128 alkyl (5.74), with a binding affinity of -8.0 kcal/mol.

SOX4

Upon investigating the structural interactions between selected compounds and *SOX4*, it was observed that dibenzo(a,e)pyrene (9126) reacted with Leu100 pi-alkyl (5.05), Pro107 pi-alkyl (5.62), Phe472 pi-pi stacked (3.69, 3.97, 4.46, 4.89, 5.32), with a binding affinity of -9.0 kcal/mol. 148413 reacted with Pro107 alkyl (4.09), pi-alkyl (5.76), Phe472 pi-pi stacked (3.74, 3.91, 3.94, 4.49, 5.26), pi-alkyl (5.48), Tyr474 pi-alkyl (4.86), with a binding affinity of -8.8 kcal/mol. 12588587with a binding affinity of -8.6 kcal/mol, reacted with Pro107 halogen (3.23, 3.92, 3.99), Arg110 pi-alkyl (5.67), halogen (3.19, 3.60), Glu111 halogen (3.80), Arg114 pi-alkyl (5.39), Ser465 H bonds (2.77, 3.08), Asn469 halogen (3.33, 3.95), Leu470 H bond (3.50), pi-alkyl (5.02). 14274984with a binding affinity of -8.5 kcal/mol, reacted with Pro107 pi-

alkyl (5.45), Phe472 pi-pi stacked (3.67, 4.06, 4.35, 5.28, 5.60), Tyr474 pi-alkyl (5.07). 23617881 reacted with Pro107 pi-alkyl (5.19, 5.30), Phe108 pi-alkyl (5.60), Phe472 pi-pi stacked (3.71, 3.97, 4.58, 4.66, 4.71, 5.60), with a binding affinity of -9.1 kcal/mol. 23621448 reacted with Leu100 pi-alkyl (5.16), Phe472 pi-pi stacked (3.71, 4.12, 4.60, 5.94), pi-alkyl (4.61), with a binding affinity of -9.1 kcal/mol. 101392782with a binding affinity of -9.0 kcal/mol, reacted with Pro107 pi-alkyl (5.60), Phe108 pi-alkyl (5.77), Phe472 pi-pi stacked (3.71, 3.84, 4.92), pi-alkyl (5.85). 129628257with a binding affinity of -9.0 kcal/mol, reacted with Pro107 pi-alkyl (5.55, 5.82), Val471 alkyl (5.77), Phe472 pi-pi stacked (3.78, 4.18, 4.55), pi-alkyl (5.87). 129716757with a binding affinity of -8.9 kcal/mol, reacted with Trp69 H bond (2.09), Lys76 H bonds (2.84, 2.97), Phe472 pi-pi stacked (3.76, 3.88, 4.15, 4.93). Lastly, 129853608 participated in bonding interactions with Ile72 pi –alkyl (5.39), Lys76 pi-cation (4.23), Leu100 pi-alkyl (5.43, 5.68), Asp104 pi-anion (3.80), Pro107 alkyl (5.47), Phe108 pi-pi t-shaped (5.05), pi-alkyl (5.44), Phe472 pi-alkyl (5.15), with a binding affinity of -8.6 kcal/mol.

ZNF3

A detailed analysis of the structural interactions between selected compounds and *ZNF3* revealed that dibenzo(a,e)pyrene (9126) with a binding affinity of -10.1 kcal/mol, reacted with Arg62 pi-alkyl (5.46), Trp65 pi-pi stacked (3.67, 3.80, 3.81, 4.08, 4.15, 4.20, 4.62, 4.81, 4.95), Arg77 pi-cation (3.61), Phe126 pi-pi t-shaped (5.55). 160249with a binding affinity of -9.9 kcal/mol, reacted with Arg62 pi-alkyl (5.07), Trp65 (3.66, 3.77, 3.80, 3.90, 4.07, 4.17, 4.57, 4.87, 5.13), pi alkyl (5.23, 5.39), Arg77 pi-alkyl (5.34), pi-cation (4.31), Phe126 pi alkyl (5.52). 169380with a binding affinity of -10.3 kcal/mol, reacted with Arg62 pi-alkyl (5.12), Trp65 (3.70, 3.79, 3.84, 3.85, 4.07, 4.17, 4.63, 4.83, 4.84, 4.95, 5.14) pi-alkyl (4.79), Arg77 pi-alkyl (5.26), Phe126 pi-alkyl (4.14). 12588587 reacted with Trp65 pi-pi t-shaped (5.33), Tyr76 H bonds (2.82, 3.01), Arg77 pi-alkyl (5.72), halogen (3.50), Leu81 pi-alkyl (5.73), Met80 pi-alkyl (5.10, 5.15), halogen (3.49), with a binding affinity of -10.3 kcal/mol. 142749984 reacted with Arg62 pi-alkyl (5.15), Trp65 pi-pi stacked (3.72, 3.78, 3.84, 3.84, 4.07, 4.19, 4.66, 4.80, 4.89, 4.99, 5.21), Tyr76 pi-alkyl (4.96), Arg77 pi-alkyl (5.32), alkyl (4.06), pi-cation (4.31), with a binding affinity of -10.3 kcal/mol. 23617881with a binding affinity of -10.2 kcal/mol, reacted with Trp65 pi-pi stacked (3.79, 3.91, 3.91, 4.06, 4.32, 4.75, 4.80, 4.84, 4.93, 5.76), Tyr76 pi-pi t-shaped (5.24), Arg77 pi-alkyl (5.24, 5.80), alkyl (4.75), Phe126 pi-pi t-shaped (5.20). 23621448with a binding affinity of -10.2 kcal/mol, reacted with Trp65 pi-pi stacked (3.85, 3.91, 4.15, 4.31, 4.36, 4.66, 4.92, 5.00, 5.41), Tyr76 pi-pi t shaped (4.92), Arg77 pi-alkyl (5.41, 5.52), Met80 pi-alkyl (5.61), Phe126 pi-alkyl (4.87), pi-pi t-shaped (5.17). 101392782 reacted with Tyr84 pi-alkyl (5.68), Val87 alkyl (5.95), Leu112 pi-alkyl (5.18, 5.25), pi-sigma (3.42, 3.99), Leu113 alkyl (4.95), Arg115 pi-alkyl (5.06), with a binding affinity of -10.3 kcal/mol. 129701154with a binding affinity of -9.9 kcal/mol, reacted with Arg62 pi-alkyl (5.11), Trp65 pi-pi stacked (3.67, 3.75, 3.82, 3.88, 4.07, 4.15, 4.59, 4.79, 4.87,

4.90), pi-alkyl (5.83), Arg77 alkyl (4.66). Lastly, 129716757 participated in bonding interactions with Arg62 pi-alkyl (5.25), Trp65 pi-pi stacked (3.77, 3.78, 3.79, 3.99, 4.12, 4.22, 4.70, 4.82, 5.48), Arg77 pi-alkyl (5.36), pi-cation (4.41), with a binding affinity of -9.9 kcal/mol.

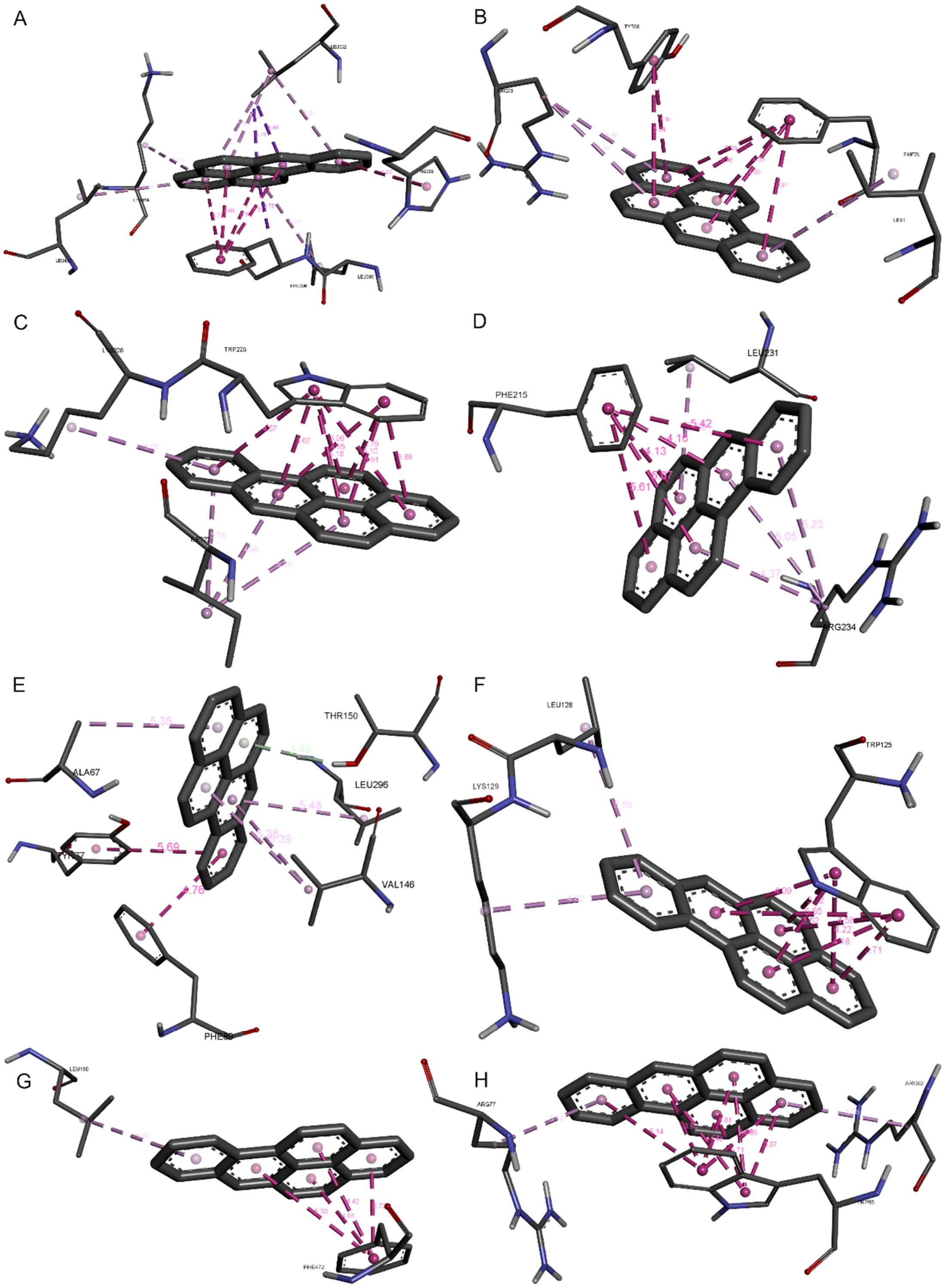

Supplementary Figure S1. Best results of benzo[a]pyrene for ADAMTS8 and its co-dependent genes (A. ADAMTS8, B. DNAL4, C. EVI2B, D. PPP1R35, E. PTGR3, F. RPL21, G. SOX4, H. ZNF3)

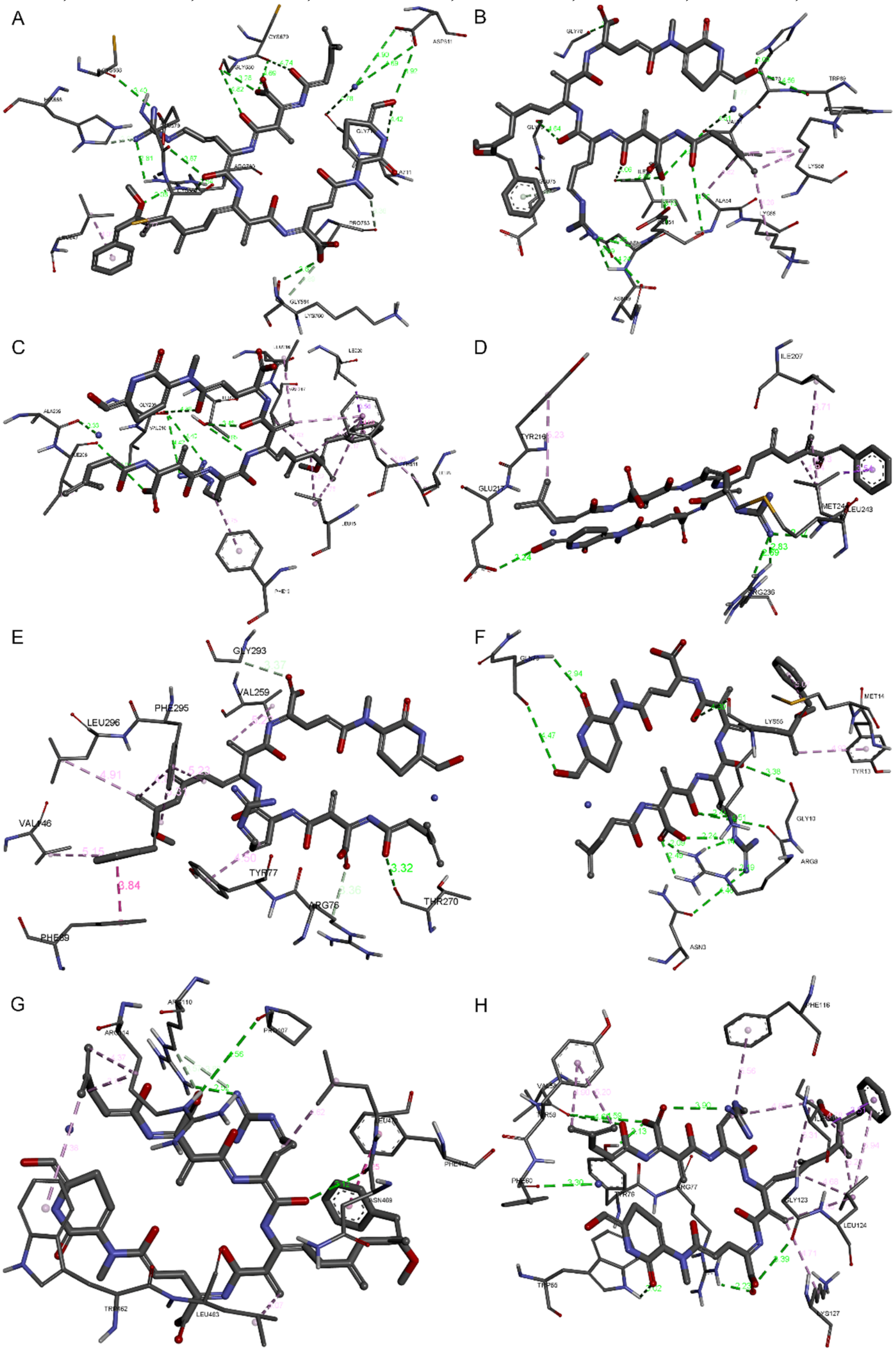

Supplementary Figure S2. Best results of cyanoginosin LR for ADAMTS8 and its co-dependent genes (A. ADAMTS8, B. DNAL4, C. EVI2B, D. PPP1R35, E. PTGR3, F. RPL21, G. SOX4, H. ZNF3)

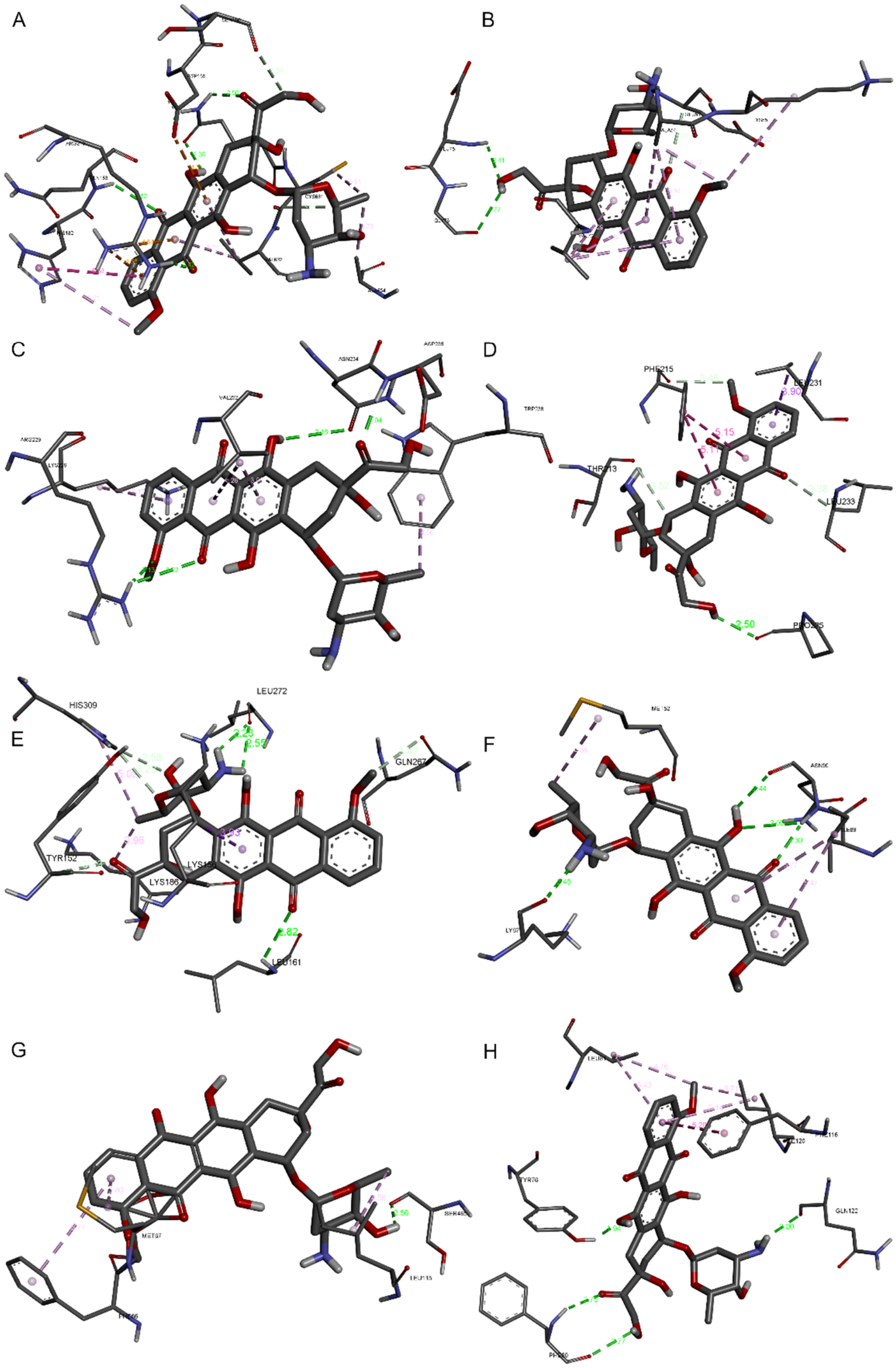

Supplementary Figure S3. Best results of doxorubicin for ADAMTS8 and its co-dependent genes (A. ADAMTS8, B. DNAL4, C. EVI2B, D. PPP1R35, E. PTGR3, F. RPL21, G. SOX4, H. ZNF3)

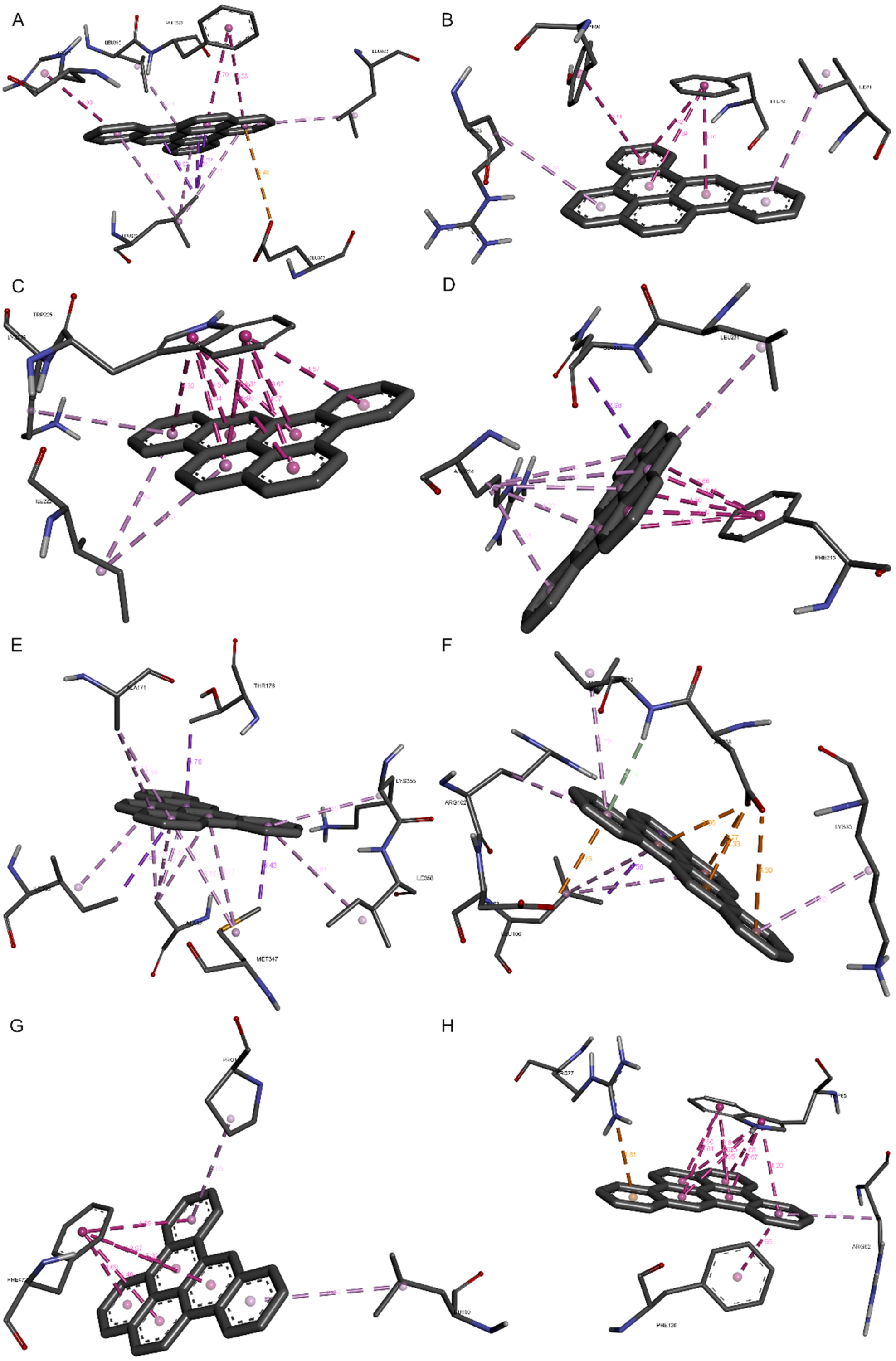

Supplementary Figure S4. Best results of dibenzo(a,e)pyrene (9126) for ADAMTS8 and its co-dependent genes (A. ADAMTS8, B. DNAL4, C. EVI2B, D. PPP1R35, E. PTGR3, F. RPL21, G. SOX4, H. ZNF3)
